# Schwannomatozis of the Chest Wall: FDG PET Findings

**DOI:** 10.4274/mirt.353

**Published:** 2014-06-05

**Authors:** Seyfettin Ilgan, Erkan Dikmen, Cihan Genco Çetinkanat, Mehmet Dakak, Adem Güngör

**Affiliations:** 1 Güven Hospital, Department of Nuclear Medicine, Ankara, Turkey; 2 Güven Hospital, Department of Thoracic Surgery, Ankara, Turkey; 3 TOBB Mesa Hospital, Department of Thoracic Surgery, Ankara, Turkey

**Keywords:** Neurilemmoma, schwannoma, chest wall, 18F-FDG, Positron-emission tomography

## Abstract

Authors present a rare case of bifocal schwannoma of thoracic wall showing metabolic activity on FDG PET images. A 43-year-old male with palpable mass on lateral chest wall was referred for F-18 FDG PET/CT imaging for differential diagnosis. His medical history and basic laboratory results were unremarkable. PET/CT images revealed a mild FDG uptake in the solid soft tissue mass in the left lateral chest wall that was growing outside of the thorax. It was located in 7th intercostal space and adjacent ribs were intact. Incidentally, second mass lesion was detected in the right posterior seventh intercostal space protruding to the pleural space and showing higher FDG uptake on PET/CT images. The lesions were surgically removed at the same session for definite diagnosis and treatment. Histopathologic evaluation of both lesions revealed benign schwannoma.

## Abstract (tr)

Toraks duvarında bifokal schwannomalı nadir bir olguda FDG PET bulguları sunulmuştur. Göğüs duvarı lateralinde ele gelen kitle nedeniyle araştırılan 43 yaşında erkek hasta ayırıcı tanı amacıyla FDG PET/BT görüntülemesi için refere edildi. Hastanın tıbbi öyküsü ve laboratuvar testleri ayırıcı tanı için özellik göstermiyordu. PET/BT görüntülerinde sol göğüs duvarında 7. interkostal aralıkta toraks dışına doğru büyüyen ve çevre kotlarda destrüksiyon oluşturmayan kitle lezyonunda hafif düzeyde FDG tutulumu izlendi. Tesadüfen, sağ göğüs duvarı posteriorunda plevral boşluğa doğru büyüyen ve görece olarak daha yüksek metabolik aktivite gösteren ikinci bir kitle lezyonu tespit edildi. Kitleler tedavi ve kesin tanı amacıyla aynı seansta cerrahi olarak çıkartıldı. Her iki lezyonun histopatolojik incelemesi benign schwannoma olarak tespit edildi.

## INTRODUCTION

Benign tumors of thoracic wall are uncommon lesions and may originate from bone, cartilage, blood vessels, fat or nerves. Schwannomas, also known as neurilemomas or neurinomas are slow-growing, encapsulated neurogenic tumours and are usually found as a solitary lesion in the extremities, and head and neck regions. Chest wall schwannoma is rare and usually found as asymptomatic solitary lesions growing towards the pleural space in the posterior mediastinum. They arise from spinal nerve roots and intercostal nerves and typically occur in patients between the second to fifth decades. Most neural tumors of the chest arise in the mediastinum; fewer than 10% of originate peripherally from intercostal nerves. Surgical treatment is indicated only if they are symptomatic are bulky or exhibit rapid growth. On the other hand, malignant schwannomas are high-grade sarcomas that most commonly arise in young and middle-aged adults. These tumors are observed as a fast growing mass, mainly in the trunk and proximal parts of the extremities. Though quite rare it has a tendency for systemic spread, especially to the lungs, which ultimately causes death in the majority of patients (1,2). Authors present a rare case of bifocal schwannoma of the thoracic wall showing metabolic activity on FDG PET images. 

## CASE REPORT

**Figure 1:**
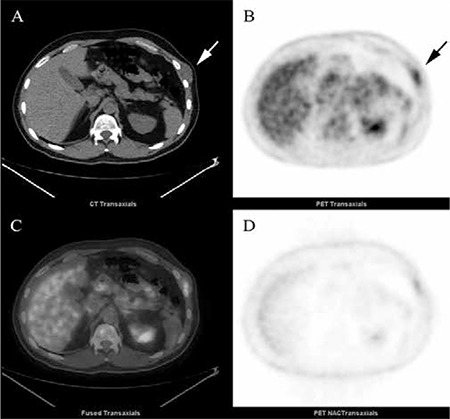
Transaxial (a) CT, (b) PET, (c) PET/CT fusion and (d) Non-attennuation corrected (NAC) PET images show a mildly hypermetabolic ovoid mass lesion (SUVmax: 3.1) in the left 7th intercostal space that is slightly hypodense compared to chest wall muscle (arrows) on non-contrast CT and 32x21 mm in size.

A 43-year-old male with slowly growing, painless palpable mass on lateral chest wall was referred for F-18 FDG PET/CT imaging to rule out possible malignancy. His medical history and basic laboratory results were unremarkable. Following six hours of fasting (serum glucose 86 mg/dL), the patient was injected with 540.2 MBq (14.6 mCi) of F-18 FDG intravenously. Images were obtained using a dedicated GE Discovery ST integrated PET/CT camera after an hour waiting period without intravenous contrast. PET/CT images revealed a mildly hypermetabolic (SUV_max_: 3.1) mass lesion in the left lateral chest wall that was growing outside of the thorax (Figure 1). It was located in 7th intercostal space and adjacent ribs were intact. Incidentally, second mass lesion was seen in the right posterior hemithorax protruding to the 7th intercostal pleural space and showing higher FDG uptake (SUV_max_: 5.6) on PET/CT images. 

**Figure 2:**
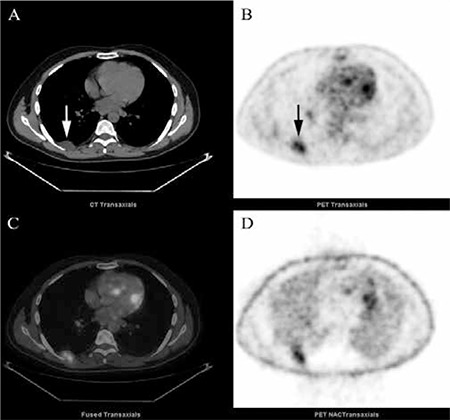
Transaxial (a) CT, (b) PET, (c) PET/CT fusion and (d) NAC PET images. A second mass lesion of the posterior chest wall in the 7th intercostal space showing moderately increased FDG uptake (SUVmax: 5.6) and 30x17 mm in size is detected (arrows).

This second mass lesion on the contralateral posterior chest wall is shown in (arrows) Figure 2. Despite similar CT characteristics, lesion was growing towards pleural space without erosion of the adjacent rib, most likely of a chest wall origin. Whole-body FDG PET images were otherwise normal. Surgical excision was recommended to rule out possible malignancy. The lesions were surgically removed at the same session for definite diagnosis and treatment. Histopathologic evaluation of both lesions revealed benign schwannoma. 

## LITERATURE REVIEW AND DISCUSSION

Non-enhanced CT appearance of schwannomas has been described as a well-circumscribed homogeneous mass of soft-tissue density with attenuation slightly less than or equal to that of muscle. Although it has been suggested that CT findings of inhomogeneities suggest malignancy, benign schwannomas can also be nonhomogeneous on CT examination (3). At MR, schwannomas have signal intensity equal to or greater than muscle on T1 weighted images. However, the radiologic features of benign and malignant chest wall tumors frequently overlap. Neither CT nor MRI are considered sufficiently accurate to differentiate benign from malignant nerve sheath tumors (3,4). 

On the other hand FDG PET could differentiate malignant peripheral nerve sheath tumors (PNST) from benign ones with high accuracy. It has been reported that a SUV_max_ threshold of 6.1 g/mL differentiated malignant from benign PNST with a sensitivity and specificity of 94% and 91%, respectively. Decreasing this threshold to 4.5 g/mL would have increased the sensitivity to 100% but reduced the specificity to 83% (5). However, FDG PET might have limited value in distinguishing schwannomas from malignant PNSTs, because reported FDG uptake by schwannomas is quite variable, with a SUV_max_ range of 1.9-12. Even SUV_max_ values as high as 6.0 cannot exclude schwannoma from the differential diagnosis (6,7,8,9). Surgical treatment still remains the standard way of diagnosis and treatment in patients with equivocal findings.
